# The Minimally Important Difference (MID) in Visual Acuity That Represents Changes in Patients’ Quality of Life

**DOI:** 10.7759/cureus.65503

**Published:** 2024-07-27

**Authors:** Osama H Ababneh, Yaqin M Alzagareet, Razan M Al-Zoubi, Dania T Ahmad, Rasha W Atieh, Aya E Odeh, Farah A Alkhaled, Saif Aldeen Alryalat

**Affiliations:** 1 Ophthalmology, Jordan University Hospital, The University of Jordan, Amman, JOR; 2 Ophthalmology, The University of Jordan, Amman, JOR

**Keywords:** minimal important difference, visual function questionnaire, visual acuity, vision-related quality of life, refractive surgery, keratoplasty, glaucoma, cataract

## Abstract

To assess the minimal change in visual acuity perceived by patients as important in different eye-related interventions. PubMed was utilized to search articles on each of the four major interventions: cataract surgery, keratoplasty, different glaucoma treatments, and refractive eye surgery, each combined with quality of life (QoL) and visual acuity keywords. The search was narrowed to articles between 2000 and 2023. Seventy-four major articles were thus reviewed. Of these, 27 studies reviewed the results of cataract surgery, 20 studies discussed the effect of keratoplasty interventions on the vision-related QoL (VRQoL), most showing that VRQoL improved significantly after keratoplasty, 11 studies investigated the effect of different glaucoma interventions on patients' visual acuity and the QoL, 16 studies reviewed refractive surgery, where they showed an improved QoL in most of the cases, although some of the studies showed a slight superiority of one intervention over the other in the short term. The minimally important difference (MID) perceived in visual acuity depends mainly on the type of surgical intervention (keratoplasty, glaucoma, or refractive surgery), and the impact on QoL on improved visual acuity differs depending on the intervention.

## Introduction and background

Visual acuity is the most common functional measure used in ophthalmology-related clinical trials and Food and Drug Administration (FDA) clinical trials for testing new eye-related treatments [1]. For studies investigating ocular diseases, the choice of an appropriate primary or secondary endpoint is particularly challenging. However, the degree of improvement in visual acuity perceived by patients as “important”, which is known as the minimally important difference (MID), is not yet agreed upon [2]. Previous studies tried to connect the change or variability in visual acuity with quality of life (QoL) measures, in order to determine the change in visual acuity that would “significantly” affect the QoL [2]. The field of QoL research is complex and heterogeneous, with significant variations between different disciplines in medicine. Ophthalmic patients are at particular risk of suffering from a reduction in QoL. However, the interrelation between QoL and visual perception is complex and cannot be reduced to a simple loss of visual acuity [2]. Nowadays, the Early Treatment Diabetic Retinopathy Study (ETDRS) visual acuity testing has become the standard for visual acuity testing and the landmark trials for intravitreal injection defined the endpoint for visual acuity improvement as 15 letters gain on the ETDRS chart [3], which has become the standard endpoint to judge meaningful difference, replacing older approaches such as Snellen and Sloan acuity tests [4]. However, it is expected that such MID visual acuity would be different between different interventions [2].

Although there is no generally accepted definition of health-related QoL, it is usually understood as a measure of a subject's well-being and/or how a certain medical condition may affect a patient on an individual level [5]. Being a subjective measure that varies between patients makes it compelling to assess each case individually, the QoL related to vision is still markedly different, so it’s important to report and recognize less-than-perfect cases to build a more robust body of literature on the topic.

In this review, we assessed the current literature on the changes in visual acuity perceived by patients as important (aka MID) in different eye-related interventions, including cataract, keratoplasty, glaucoma treatments, and refractive eye surgery, which will eventually help highlight the procedures' safety and efficacy while improving management when complications occur.

## Review

Methods

For this narrative review, PubMed was the search engine that was utilized to search articles about each of the four major ocular interventions: cataract eye surgery, keratoplasty, different glaucoma treatments, and refractive eye surgery, each combined with QoL and visual acuity keywords. The search was limited to articles published between 2000 and 2023.

The search strategy included terms related to each of the following major ophthalmic specialty-related interventions: (i) Cataract eye surgery: phacoemulsification (PE), femtosecond laser-assisted cataract surgery (FLACS), extracapsular cataract surgery (ECCE), and manual small incision cataract surgery (MSICS); (ii) Keratoplasty: penetrating keratoplasty, Descemet membrane endothelial keratoplasty (DMEK), Descemet stripping automated endothelial keratoplasty (DSAEK), deep anterior lamellar keratoplasty (DALK) and keratoprosthesis; (iii) Glaucoma treatment: micro-invasive glaucoma surgery (MIGS), trabeculectomy (TE), non-valve tube shunt surgery, glaucoma filtration surgery, Ex-PRESS (excessive pressure regulating shunt system) filtration shunt implantation, laser trabeculoplasty, topical medications, and glaucoma with PE; (iv) Refractive eye surgery: small incision lenticule extraction (SMILE), laser-assisted subepithelial keratectomy (LASEK), laser-assisted in situ keratomileusis (LASIK) with different techniques, and implantable collamer lens phakic intraocular lens (pIOL).

The search for each intervention was combined with QoL-related terms, including vision-related QoL (VRQoL), MID, and visual acuity. To avoid bias, all study types were considered acceptable for this review, with no complete exclusion of any particular type. However, there was a higher inclusion proportion for original articles compared to other types of articles.

The primary outcome was assessing changes in VRQoL and visual acuity following various eye-related interventions, encompassing cataract eye surgery, keratoplasty, diverse glaucoma treatments, and refractive eye surgery. Secondary outcomes included evaluating postoperative complications and associated costs across diverse eye-related interventions.

MID in cataract surgery

Cataract is a major cause of global blindness, accounting for 50-80% in developing countries, and this number is expected to rise due to the increase in life expectancy [6]. Cataract surgery is one of the most frequent interventions currently undertaken in medicine worldwide and it has significantly improved the lives of many people. However, perfection seems to be a moving target, and there remains room for improvement [7]. Several studies have indicated that cataract surgery generally leads to a favorable visual outcome. A multicenter database study conducted over five years in Malaysia found that among various types of cataract surgery, 92.9% of individuals achieved a postoperative best-corrected visual acuity (BCVA) better than 6/18 [8]. A hospital-based prospective cohort study carried out in Ghana assessed visual functions and patient satisfaction using the National Eye Institute Visual Function Questionnaire (NEI-VFQ 25), and concluded that overall visual functions improved significantly and patients' satisfaction was high with an average QoL score of 77.46 [9]. Several other studies assessed the impact of cataract surgery in different age groups and populations and found favorable outcomes in terms of visual acuity [10,11].

Two studies have shown that the results after PE or ECCE with intraocular lens (IOL) implantation were similar. For instance, one study in Pakistan found that over two-thirds of patients had a favorable visual outcome (that is, 6/18 or better) for both techniques after refraction [12]. In addition to that, a cross-sectional study in Guangzhou, China showed that the different visual acuity outcomes between ECCE with IOL and PE with IOL were not statistically significant and that more than half of the eyes with poor outcomes due to uncorrected aphakia, refractive errors, or posterior capsule opacity are potentially treatable [13]. However, both previous studies showed that the visual acuity outcomes with IOL implantation were significantly better than those without IOL. Other studies compared ECCE and intracapsular cataract extraction (ICCE) techniques specifically. In 2001, a survey in Ghana found that those who had ECCE with IOL surgery had a presenting visual acuity of 20/60 or better in 65% of eyes at the time of the survey, which is significantly better than the 30% in ICCE surgery [14]. They also found that 9% of the population aged 40 years or older needed cataract surgery in one or both eyes to improve vision. Supporting that, the findings of a review showed that ECCE provides better visual outcomes and has fewer complications than ICCE [11]. Another study showed that ICCE was more likely to result in visual acuity of <6/18 than ECCE with IOL [15]. On the contrary, one study in Uganda showed no significant differences in corrected or uncorrected final distance visual acuity achieved with ECCE with posterior chamber IOL versus ICCE with anterior chamber IOL after a median follow-up of 17.5 months [16]. However, where both techniques are available, ECCE with PCIOL should be the first choice because of fewer complications [16].

Other studies highlighted factors other than the type of surgery that might affect the outcomes. For example, a non-comparative case series results where most of the patients underwent MSICS showed that even though the best corrected visual acuity of 6/18 or more was achieved in 94% of the patients, factors such as age above 60 years, sex (males have less postoperative visual acuity), and surgeon experience were found to be statistically significant risk factors for outcomes [17]. In addition to that, a couple of studies highlighted the urban and rural differences in cataract surgical outcomes suggesting that rural eye camp surgeries are significantly more likely to result in visual acuity of 6/18 or less than hospital‐based surgeries [13,15]. 

Regarding postoperative complications, one study showed that the overall intraoperative and immediate postoperative complications post cataract surgery occurred in 1.9% and 12.6% of the patients, respectively where the majority of the patients underwent MSICS [17]. Another case series stated that the operative and postoperative complications were similar for both ECCE and PE [18]. Moreover, a study comparing three cataract surgeries (ECCE, PE, and MSICS) showed that a shorter corneal incision implies fewer complications, less operative trauma, faster visual rehabilitation, and a better visual outcome [19].

Numerous studies have investigated the impact of ECCE on visual acuity and its postoperative complications. A retrospective study conducted in India showed that 88.88% of patients achieved a final BCVA of 6/12 or better, and 80.19% had a final refraction with less than 2.0 diopters of astigmatism [20]. In a separate study from Oman involving 33 patients (35 eyes) who underwent ECCE with posterior chamber IOL for mature cataracts, with pseudoexfoliation, 85.71% of eyes experienced an improvement in BCVA ranging from 0.2 to 1.0 Snellen lines after one year. Four eyes (11.43%) maintained the same BCVA as the baseline, and no intraoperative complications occurred [21]. A study in Turkey evaluating the results of ECCE with IOL implantation in patients with Behçet's disease found that, in eyes with Behçet's uveitis, 21% achieved a postoperative visual acuity of 0.5 or better after a mean follow-up of 34.2±4.6 months (range: 5-66 months) and most of the patients who had low visual acuity were due to preoperatively existing optic atrophy and/or inflammatory degeneration or macular edema [22]. The most common postoperative complication observed was posterior capsule opacification, affecting 47% of cases [22]. Furthermore, the authors of a study conducted in Nepal reported the short and medium-term outcomes of a prospective series of sutureless manual ECCE procedures at a high-volume surgical center. They concluded that rapid recovery of good vision can be achieved at a low cost in regions where high-volume cataract surgery is necessary. Notably, postoperative astigmatism was identified as the primary cause of uncorrected acuity falling below 6/18 [23].

Several studies have been conducted comparing PE and ECCE. A recent systematic review and meta-analysis encompassing a total of eight articles and 1,015 affected eyes concluded that in comparison to ECCE, PE yields enhanced postoperative visual acuity and presents fewer complications [24]. This consensus was corroborated by another review article involving 11 trials and 1,228 participants [25]. A study conducted in a Malaysian district hospital revealed significantly superior visual outcomes for PE over ECCE (p=0.001) [26]. Nevertheless, no significant correlation between intraoperative complications and the type of surgery was observed [26]. Results from another randomized trial indicated the clinical superiority of PE. Surgical complications and capsule opacity within one year after surgery occurred less frequently in the PE group, with a higher proportion achieving unaided visual acuity of 6/9 or better [27]. Conversely, a separate study demonstrated that ECCE may be more suitable than PE in complex cataract surgery following penetrating keratoplasty. In the ECCE group, endothelial cell density was significantly higher than in the PE group, and the endothelial cell loss rate was notably lower at two, four, and six months post cataract surgery. However, there was no significant difference in postoperative BCVA between the two groups (P = 0.065) [28]. Another study found that ECCE with a small incision has comparable effects on post-surgery BCVA improvement to PE; therefore, ECCE could be an alternative cataract surgical treatment in economically underdeveloped areas in China, provided the surgeons are adequately trained [29].

Numerous studies have undertaken comparisons between ECCE and MSICS techniques. One observational study from South Africa concluded that the MSICS technique yielded superior post-surgery visual acuity outcomes when compared to ECCE [30]. A review of three trials (two trials were conducted in India and one in Nepal) revealed that surgically induced astigmatism was more prevalent with the ECCE procedure in two of the trials that reported this outcome. In one study, the MSICS group experienced more intra- and postoperative complications, while another study reported that the costs of the two procedures were similar [31]. In another comparative study, it was demonstrated that the rate of complications was comparable for both techniques. However, the MSICS technique offered slightly better visual and refractive outcomes due to reduced corneal astigmatism induction, and transitioning from ECCE to MSICS for experienced cataract surgeons in surgical campaigns is safe [32]. Furthermore, a clinical trial affirmed that both MSICS and ECCE are safe and effective techniques for treating cataract patients in community eye care settings. MSICS requires similar equipment to ECCE but provides superior uncorrected vision [33].

MID in keratoplasty

Many of the reviewed papers have compared the effects of different types of keratoplasties on QoL. One of these is a randomized controlled trial conducted in the Netherlands, it compared the VRQoL in patients undergoing DMEK or ultrathin DSAEK (UT-DSAEK), and the results showed that VRQoL improved significantly after surgery but did not differ between both techniques [34]. A similar conclusion was reached by the authors of two other comparative studies [35,36]. Furthermore, a separate comparative study compared the VRQoL after different keratoplasty techniques (penetrating keratoplasty, DALK, DSAEK, and DMEK) and showed that there was a significant improvement in QoL after all these different techniques after an average of 1.5 years postoperatively [37]. There were no significant differences in NEI-VFQ 9 scores between patients treated with DMEK versus DSAEK (P = 0.440) or between patients treated with penetrating keratoplasty versus DALK (P = 1.000) [37]. When controlling for postoperative acuity, VRQoL was similar among all study participants, irrespective of the keratoplasty technique [37]. On the other hand, another study compared the QoL after bilateral UT-DSAEK and bilateral penetrating keratoplasty in Fuchs' dystrophy concluded that the VRQoL indicates a higher satisfaction in UT-DSAEK eyes in terms of corrected distance visual acuity, contrast sensitivity, together with less higher-order aberrations [38]. This was supported by a different study comparing DSAEK, penetrating keratoplasty, and keratoprosthesis which showed that patients treated with penetrating keratoplasty (P = 0.002) or keratoprosthesis (P = 0.019) were found to have poorer QoL scores than those treated with DSAEK [39]. They also found that older recipients' age and better baseline vision seem to be associated with an improved QoL [39]. A different study showed that VRQoL in patients with Fuchs' endothelial dystrophy is significantly impaired but improves after keratoplasty irrespective of the technique, but the improvement is faster after DSEK than after penetrating keratoplasty [40]. A review article conducted in 2006 concluded that most of the effects of changing techniques are small, and there was no evidence for the superiority of any specific technique in terms of improved QoL or cost-effectiveness [41]. A retrospective study used another type of comparison and showed that penetrating keratoplasty for patients with severe keratoconus seems to be a comparatively effective and cost-effective procedure when compared to other interventions across different medical specialties [42].

Visual Function Questionnaire (VFQ) 25, a tool in which the higher the score, the better the QoL, has been used by many researchers in the reviewed papers, one of those concluded that VRQoL improved significantly after DALK and continued to improve after suture removal [43]. Another paper reached a similar conclusion and pointed out that bilateral endothelial keratoplasty resulted in higher VRQoL compared to unilateral cases [44]. Another study, in which the subjective assessment of visual quality was evaluated using the NEI-VFQ concluded that DSAEK may restore corneal clarity and increase the VRQoL [45]. VFQ 39 (VFQ-39) and a straylight questionnaire have been used by another article showing that the quality of vision after manual DSEK does not return to normal levels in age-matched pseudophakic eyes, hence, questionnaire scores indicate mild (VFQ 39) to moderate (straylight) subjective visual impairment [46]. Another study used the SF-12 Health Survey, before and one year after surgery, and a six-item questionnaire on satisfaction with graft outcomes showed that grafting improves patients' health-related QoL, influencing mental health (i.e., psychological attitude, social interaction, and emotions) with minor effects on physical health (limitation, pain, and vitality) [47].

This was also proven by a study from Russia that showed a significant increase in visual acuity in keratoconus patients who underwent intrastromal keratoplasty, with improvement in general health, and a more optimistic view of one's future [48]. A multicenter study conducted in the United States also showed that improvement in visual function is inversely associated with visual acuity in the better-seeing eye but does not correlate with postoperative acuity in the grafted eye [49]. A study conducted in Germany showed that VRQoL improves similarly after DMEK and triple-DMEK and between the first and second operated eye, and the extent of improvement is independent of the preoperative BCVA [50]. Furthermore, a study conducted in France showed that DMEK provides good visual rehabilitation and an improvement in QoL for patients with Fuchs' endothelial dystrophy despite having higher-order optical aberrations in these patients compared to a healthy population [51].

A prospective study aimed to assess the safety and efficacy outcomes of sutured custom silicone artificial iris and IOL implantation combined with penetrating keratoplasty and showed that the procedure was effective at improving corrected distant visual acuity, cosmesis, and QoL; however, it was associated with frequent postoperative complications, of which iritis, intraocular pressure (IOP) elevation, and secondary graft failure were the most common [52]. A comparison between DMEK and microthin (MT)-DSAEK was conducted using a randomized controlled study and showed that patient satisfaction was similar with no differences reported in VRQoL scores, as was the complications profile between both groups. However, DMEK surgery resulted in significantly better BCVA at one, three, six, and 12 months postoperatively compared with MT-DSAEK [53].

MID in glaucoma

Several studies were done to compare different approaches that can be used for treating glaucoma. For example, a multicenter randomized clinical trial compared the efficacy of non-valve tube shunt surgery (Baerveldt glaucoma implant) to trabeculectomy with mitomycin C (MMC) 0.4 mg/ml for four minutes in patients with previous intraocular surgery (previous trabeculectomy, cataract extraction with IOL implantation, or both). The Tube Versus Trabeculectomy (TVT) study concluded that after five years of follow-up, tube shunt surgery had a higher success rate compared to trabeculectomy with MMC. Both procedures were associated with similar IOP reduction and use of supplemental medical therapy at five years [54]. They also found that additional glaucoma surgery was needed more frequently after trabeculectomy with MMC than tube shunt placement. Another study was designed to compare the effectiveness of three different categories of glaucoma treatment strategies. The incisional surgery group had the greatest rate of patients who reported an improvement in at least two lines of vision (14.2% compared with 9.9% for laser surgery and 10.9% for additional medication) [55]. For the Glaucoma Symptom Scale or NEI-VFQ measures or subscales, no clinically significant differences were observed between the benefit for the laser surgery or incisional surgery groups compared with additional medications [55].

Another comparative study was conducted to assess the impact on the QoL of MIGS (iStent, Trabectome) and a penetrating technique such as trabeculectomy. The results showed that there was no significant difference between trabeculectomy and MIGS in the QoL six months postoperatively [56]. IOP was significantly lower in trabeculectomy compared to MIGS at six weeks and three months postoperatively as well and they needed lower numbers of topical medication in the trabeculectomy group, which would impact QoL even though it is not included in the NEI-VFQ-25 [56]. In addition to that, an experimental study was conducted to observe the short-term efficacy of Ex-PRESS filtration shunt implantation in the treatment of open-angle glaucoma and the results showed that the success rate of Ex-PRESS filtration shunt implantation after the three-month follow-up is equivalent to that of classic trabeculectomy with better anterior chamber stability. There was no significant difference in VRQoL between both groups [57].

Four studies were done to compare selective laser trabeculoplasty (SLT), primary trabeculectomy, and topical medications as an initial glaucoma treatment. The first one compared SLT and topical medications. Ultimately, the study showed no proof that SLT was more effective than medication at enhancing QoL. Even though the pharmaceutical group's IOP reduction was superior to the SLT group after 24 months ((p=0.022) higher in the medication compared with the SLT group), these patients had more eyelid erythema and conjunctival hyperemia overall [58]. The second study was done to determine whether primary trabeculectomy is superior to primary medical treatment for newly diagnosed advanced glaucoma, showed that primary trabeculectomy had similar QoL and safety outcomes and achieved a lower IOP compared with primary medications (Mean IOP was 12.4 mmHg for trabeculectomy and 15.1 mmHg for medical management (mean difference −2.8 mmHg; P<0.001) [59]. The third study was held in China, and compared patients with primary open-angle glaucoma and ocular hypertension in response to SLT in relation to the QoL [60]. Participants in this trial were younger, more myopic, and had more severe visual field defects, and the difference between ocular hypertension and primary open-angle glaucoma on the EQ-5D-5L (EuroQol EQ-5D-5 levels) in the Chinese value version was 0.024 (95%CI 0.01 to 0.04) [60], compared to The United Kingdom Laser in Glaucoma and Ocular Hypertension (LiGHT) trial [61]. The fourth study published in 2001 used all available follow-up data from the collaborative initial glaucoma treatment five years after the start of treatment [62]. Over time, there was a general decrease in the percentage of participants reporting symptoms in both therapy groups. The surgical patients reported trouble with local eye symptoms. However, there were very few treatment-group differences in visual functioning.

Other studies were conducted to assess the effectiveness of a single approach rather than comparing different approaches. One study was done to evaluate the VRQoL following glaucoma filtration surgery and it showed that although the surgery by itself did not decrease the VRQoL in glaucoma patients, there was a significant improvement in the VRQoL after the patients underwent combined cataract and glaucoma filtration surgery [63]. Another study was done to assess the effect of PE surgery on various parameters in patients with glaucoma when the cataract is of significant density. It revealed that PE cataract surgery was associated with a significant improvement in all domains of the visual function questionnaire, a decrease in the number of anti-glaucoma medications, and IOP together with deepening of anterior chamber and better QoL in patients with co-existing glaucoma [64].

MID in refractive eye surgery

Several studies have examined the impact of refractive eye surgery on the quality of patients’ lives. A prospective, consecutive series study was published in 2019 to compare QoL outcomes following ReLEx SMILE or Femto-LASIK procedures and the results showed a higher satisfaction trend and long-term QoL in patients undergoing ReLEx SMILE in comparison to Femto-LASIK in the short term in the treatment of myopic astigmatism [65]. However, Long-term results demonstrated high patient satisfaction with both methods and the overall QoL indicators were statistically significant (P<0.01) exceeding preoperative values one month after the operation [65]. In addition, a prospective, comparative, non-randomized clinical study comparing post-refractive dry eye disease after SMILE versus LASIK concluded that the SMILE procedure was less associated with dry eye disease compared to LASIK and subsequent degradation in QoL [66]. However, two studies found no statistical difference between LASIK and SMILE surgeries on QoL: one was a prospective study conducted at the Singapore National Eye Centre [67], and the other was a cross-sectional study [68], which showed that postoperative QoL is similar after, although, dry eye symptoms and glare were milder in the SMILE group.

One of the supportive studies of bilateral wavefront-guided LASIK was a prospective case series study that concluded that wavefront-guided LASIK not only afforded clinically measurable improvements in vision but also significant improvements in VRQoL one year after surgery [69]. In addition to that, a prospective, non-comparative, multicenter study was conducted to evaluate the outcomes of wavefront-guided LASIK for the correction of myopia and concluded that the treatment was safe, effective with excellent visual and refractive outcomes, high patient satisfaction, and improved QoL [70]. Another retrospective case series aimed to compare the VRQoL five years after pIOL implantation with wavefront-guided LASIK for myopia. They found that pIOL implantation may offer significant VRQoL advantages (e.g., fewer activity limitations and symptoms) in the long term [71]. In another study, the QoL Impact of Refractive Correction (QIRC) questionnaire was used to conclude that Femto-LASIK leads to marked improvements in refractive error-related QoL [72].

A prospective comparative study aimed to compare preoperative contact lens trial of monovision treatment with a bilateral hyperopic presbyopic LASIK (Custom-Q mode) technique. The conclusion was that after six months post LASIK, 100% of patients achieved 20/20 or better binocular uncorrected distant visual acuity versus 57% in the monovision trial group, 100% of patients achieved 20/25 or better binocular uncorrected intermediate visual acuity versus 32% in the monovision group, while 92.86% of patients achieved 20/25 or better binocular uncorrected near visual acuity in both groups [73]. However, people receiving LASEK were less likely to achieve a refractive error within 0.5 diopters of the target at the 12-month follow-up [74].

In addition to that, a retrospective case series study was conducted to assess VRQoL outcomes five years after LASIK surgery. In conclusion, patient-reported QoL rates remained high five years after LASIK and uncorrected vision was the strongest predictor of satisfaction [75]. Furthermore, a prospective hospital-based study highlighted that LASIK may even improve mental health-related QoL [76]. Also, we came across a case series reporting long-term follow-up of two pediatric patients who underwent LASIK for anisometropic myopic amblyopia in 1999, and 16 years after their initial procedure they were clinically assessed with visual acuity testing, refraction, and QoL assessment. The two patients had stable corrected distance visual acuity, balanced refraction, improved stereopsis, and good VRQoL. Corneal topography showed a mildly decentered ablation bed with no evidence of ectasia [77].

Two studies employed the Patient-Reported Outcomes with LASIK (PROWL) questionnaire. One prospective observational study aimed to assess satisfaction, eye-related symptoms, and their effect on functioning and well-being following LASIK, and it showed that measures improved from baseline to follow-up [78]. Hours of work did not change significantly from baseline to one-month follow-up. The other study by Eisenbeisz et al. highlighted the psychological aspect of corneal refractive surgeries in three cases of completed or attempted suicide after poor refractive surgery outcomes were discussed [79]. It was found that dry eye symptoms are the most common complication after LASIK but the overall patient satisfaction rate after surgery remains around 95% with around one million surgeries performed in the United States each year. They concluded that patients would continue to pursue refractive surgery with high expectations; however, prevention by proper screening paired with early treatment may help minimize the effect of complications [79].

On the other hand, one case report showed a deterioration in the QoL after refractive surgery. It is a case report of a 34-year-old female patient who underwent LASIK surgery at the age of 24 years but experienced dissatisfaction with the outcome. Although her ocular parameters were normal after the surgery, there was no effect on her sense of pain; she reported experiencing severe eye strain, subjective dry eye symptoms, and lower QoL after the surgery with loss of her strength and self-confidence. Her symptoms improved after strength therapy [80].

Limitations

The main limitation of this study is the non-systemic nature of the methodology, which aims to describe the current literature for different ophthalmic interventions and their association with QoL. The other limitation is the lack of prospective, controlled studies that compare the superiority of one surgical intervention over others.

## Conclusions

Different ophthalmic procedures had a favorable impact on QoL regardless of their visual acuity impact. However, future studies should quantitively assess such impact in order to use customized visual acuity improvement measures for different ophthalmic interventions. Some ophthalmic procedures may prove useful in different situations and countries given the cost of the procedure and the high demand for that particular procedure. The QoL generally improved in patients undergoing ophthalmic procedures with low complication rates.
